# Spatiotemporal modeling and prediction of soil heavy metals based on spatiotemporal cokriging

**DOI:** 10.1038/s41598-017-17018-5

**Published:** 2017-12-01

**Authors:** Bei Zhang, Yong Yang

**Affiliations:** 1 College of Resources and Environment, Huazhong Agricultural University, Wuhan, 430070 China; 2 Key Laboratory of Arable Land Conservation (Middle and Lower Reaches of the Yangtze River), Ministry of Agriculture, Wuhan, 430070 China

## Abstract

Soil heavy metals exhibit significant spatiotemporal variability and are strongly correlated with other soil heavy metals. Thus, other heavy metals can be used to improve the accuracy of predictions when performing spatiotemporal predictions of soil heavy metals within a given area. In this study, we propose the spatiotemporal cokriging (STCK) method to enable the use of historical sampling points and co-variables in the spatial prediction of soil heavy metals. Moreover, experimental spatiotemporal (ST) semivariogram and ST cross-semivariogram computational methods, a fitting strategy to the ST semivariogram and ST cross-semivariogram models based on the Bilonick model, and the STCK interpolation algorithm are introduced; these methods are based on spatiotemporal kriging (STK) and cokriging (CK). The data used in this study consist of measurements of soil heavy metals from 2010 to 2014 in Wuhan City, China. The results show that the behavior of predictions of the concentrations of heavy metals in soils is physically more realistic, and the prediction uncertainties are slightly smaller, when STCK is used with greater numbers of co-variables and neighboring points.

## Introduction

Soil plays a very important role in the food chain and hence is a key pathway through which humans come into contact with most pollutants^1^. This statement is especially true for heavy metals, which have also been identified as co-factors in many diseases^2,3^. Therefore, there is considerable interest in the best way to monitor soil quality to ensure that soil is managed sustainably^4^. In recent years, an increasing amount of concern has been directed toward the spatial distribution of soil contamination^5–7^. Previous studies have carried out multivariate analyses, analyses of various pollutant indices, and geostatistical analyses to evaluate the degree of soil pollution by heavy metals using sampling data collected during individual periods. Additionally, some researchers have begun to address the concern over the spatiotemporal (ST) variability in soil heavy metals^4,8^ and have performed statistical analyses of data collected during field surveys conducted in different years that were performed to characterize the spatial and temporal changes in the concentrations of heavy metals in soils. However, the sampling and analysis procedures used when the status of soil heavy metals within a given area must be continuously monitored are expensive and time-consuming. Therefore, spatiotemporal interpolation is necessary because it enables the use of previous soil sampling points to predict present-day spatial distributions with fewer soil samples.

Spatiotemporal kriging (STK) is a tool that is used to analyze and map ST phenomena using point observations^9,10^. The technique is currently used in many research problems and fields, such as the interpolation of soil water and salinity content^11–13^, climatology^14^, and air quality monitoring^15^. Most studies use only measurements of the variable of interest. However, after soil samples have been obtained, various heavy metals in the soil can be measured simultaneously. In addition, many studies indicate that correlations exist among the various heavy metals found in soils^16–18^. The use of such relationships in interpolation via cokriging (CK) may decrease prediction uncertainties^19^.

Based on the above discussion, we believe that it is possible to combine historical sampling points and co-variables to perform predictions of heavy metals. Thus, we propose a spatiotemporal cokriging (STCK) method that is based on STK and CK. The main objective of this study is to predict the ST distribution of soil heavy metals within a study area using STCK. To achieve this objective, the following steps are followed. (1) The methods of obtaining experimental ST semivariograms and ST cross-semivariograms are explored. (2) Models for experimental ST semivariograms and ST cross-semivariograms are fitted. (3) An algorithm for STCK interpolation is proposed. Finally, (4) the accuracy and uncertainty of STCK given different combinations of co-variables and different neighboring points are explored.

## Materials

### Study area

The study area lies to the east of the Qingshan district (latitude 30°37′N, longitude 114°26′E) of Wuhan City, which is the capital of Hubei Province and the largest city in the middle reach of the Yangtze River in China. Since the 1950s, this district has seen considerable industrialization, and it currently contains 133 industrial enterprises above a designated size (with annual business incomes more than 20 million RMB, or approximately 3.3 million U.S. dollars). Some of these industrial enterprises, such as the Wuhan Iron and Steel Corporation, the China First Metallurgical Construction Co., Ltd., and the Wuhan Heavy Casting and Forging plant, are very large and include heavy industries. The land east of this region is used to plant crops and vegetables, such as rice, eggplant, cabbage, cayenne pepper, and other common Chinese vegetables. The history of planting in this area is approximately 30 to 40 years long.

### Sample collection and analysis

**Figure 1:**
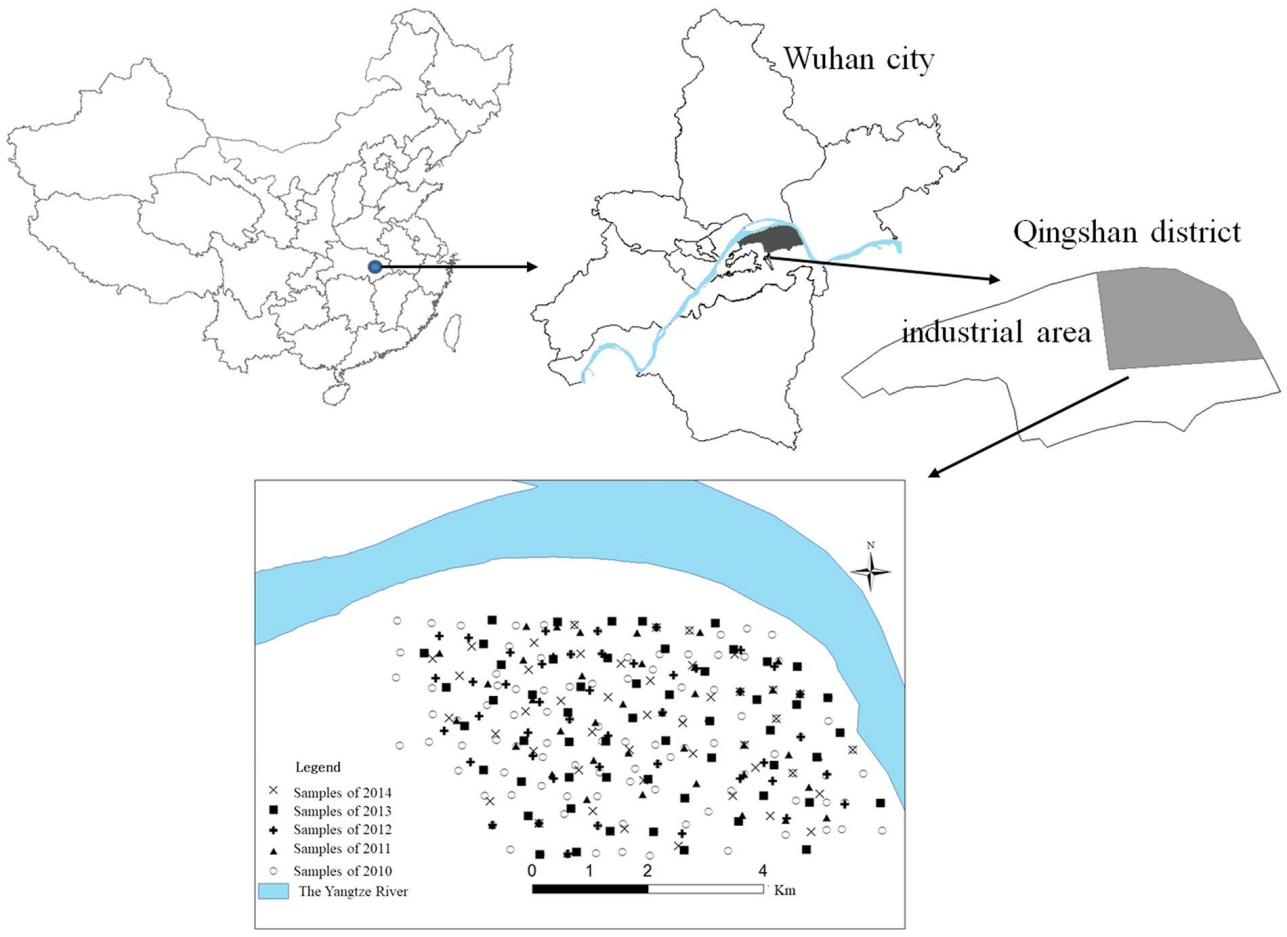
Location of the study area and the spatial distribution of soil 86 sampling points during 2010–2014 (created 87 using ArcMap, version 10.2; http://www.esri.com/).

An extensive investigation of the soil within the study area was carried out in October 2010. In total, 124 topsoil samples were collected at depths of 0–20 cm within the study area. We found that the soil pollution in this area was serious. To monitor the degree of soil contamination, we collected topsoil samples from the study area in October from 2011 to 2014. Forty-five, 48, 55, and 48 soil samples were collected in 2011, 2012, 2013 and 2014, respectively. The spatial distribution of soil sampling points are shown in Fig. 1. At each sampling point, 5 sub-samples were collected at random and mixed to obtain a composite soil sample. Any foreign debris present in the soil samples was manually removed during sample collection. The coordinates of the sample locations were recorded with a GPS. All of the soil samples were air-dried at room temperature and passed through a 100-mesh nylon sieve, which included 100 holes within an area of 1 square inch. The prepared soil samples were then stored in polyethylene bottles for analysis.

The concentrations of heavy metals, including copper (Cu), cadmium (Cd), lead (Pb), and zinc (Zn), were measured in the soil samples. Approximately 0.5 g of each prepared soil sample was digested using a mixture of nitric acid (HNO_3_) and perchloric acid (HClO_4_) in a Teflon beaker on a hot plate. The total concentrations of Cd, Cu, Cr, Pb and Zn in the digested solutions were measured using inductively coupled plasma mass spectrometry (ICP-MS; TMO, USA). The accuracy and precision of the measurements were tested using standard reference materials (GGS-3) obtained from the National Center for Standard Reference Materials of China. All of the soil samples were analyzed at the Key Laboratory of Arable Land Conservation (Middle and Lower Reaches of the Yangtze River) at the Ministry of Chinese Agriculture.

## Methods

### Spatiotemporal Cokriging

To begin, we consider two ST variables, *Z*_*u*_(x) and *Z*_*v*_(x), which we denote *u* and *v*, respectively; both of these variables obey the intrinsic hypothesis. To distinguish between space and time, let *Z*(x) = {*Z*(***s***, *t*)|***s*** ∈ ***S***, *t* ∈ *T*} be a variable that is defined on a geographical domain ***S*** ∈ *R* ^2^ and a time interval *T* ∈ R. The aim of this study is to predict the attribute *u* at a spatiotemporal point (***s***_0_, *t*_0_) where *u* was not measured. The prediction is based on measurements of *u* and *v* at *n* ST points (***s***_*i*_, *t*_*i*_), i = 1… n. Note that not all *u* and *v* are observed at the same ST points; however, some ST points where *u* and *v* can be measured are required.

Under appropriate stationarity assumptions, an estimate of the ST semivariogram may be obtained from the measurements by computing the experimental semivariogram ${\hat{\gamma }}_{uu}({h}_{{\boldsymbol{S}}},{h}_{T})$, ${\hat{\gamma }}_{vv}({h}_{{\boldsymbol{S}}},{h}_{T})$ and the cross-semivariogram ${\hat{\gamma }}_{uv}({h}_{{\boldsymbol{S}}},{h}_{T})$:

1 $${\hat{\gamma }}_{uu}({h}_{{\boldsymbol{S}}},{h}_{T})=\frac{1}{2{N}_{u}({h}_{{\boldsymbol{S}}},{h}_{T})}\sum _{i=1}^{{N}_{u}({h}_{{\boldsymbol{S}}},{h}_{T})}{[{z}_{u}({\boldsymbol{s}},t)-{z}_{u}({\boldsymbol{s}}+{h}_{{\boldsymbol{S}}},t+{h}_{T})]}^{2}$$

2 $${\hat{\gamma }}_{vv}({h}_{{\boldsymbol{S}}},{h}_{T})=\frac{1}{2{N}_{v}({h}_{{\boldsymbol{S}}},{h}_{T})}\sum _{i=1}^{{N}_{v}({h}_{{\boldsymbol{S}}},{h}_{T})}{[{z}_{v}({\boldsymbol{s}},t)-{z}_{v}({\boldsymbol{s}}+{h}_{{\boldsymbol{S}}},t+{h}_{T})]}^{2}$$

3 $$\begin{array}{rcl}{\hat{\gamma }}_{uv}({h}_{{\boldsymbol{S}}},{h}_{T}) & = & \frac{1}{2{N}_{uv}({h}_{{\boldsymbol{S}}},{h}_{T})}\sum _{i=1}^{{N}_{uv}({h}_{{\boldsymbol{S}}},{h}_{T})}\{[{z}_{u}({\boldsymbol{s}},t)-{z}_{u}({\boldsymbol{s}}+{h}_{{\boldsymbol{S}}},t+{h}_{T})]\\  &  & \times [{z}_{v}({\boldsymbol{s}},t)-{z}_{v}({\boldsymbol{s}}+{h}_{{\boldsymbol{S}}},t+{h}_{T})]\}\end{array}$$

where *h*_***S***_ and *h*_*T*_ are the ***S*** and *T* lags, respectively, and *N*_*u*_(*h*_***S***_, *h*_*T*_), *N*_*v*_(*h*_***S***_, *h*_*T*_), and *N*_*uv*_(*h*_***S***_, *h*_*T*_) are the numbers of pairs in the ST lag for *u*, *v* and *uv*, respectively.

Fitting the models to the ST experimental semivariogram and cross-semivariogram has some additional problems over conventional semivariogram and cross-semivariogram modeling; these problems arise due to the distinct differences between the variations in *S* and *T* ^10^. In this study, we use an extension of the separate-sum models proposed by Bilonick^19^, in which geometric and zonal anisotropy are applied to the problems arising from the differences in *S* and *T* variability. In the Bilonick model, the semivariogram is divided into three parts: an *S* part, a *T* part and an ST part that includes only geometric anisotropy and neglects zonal anisotropy. Assuming that these three parts are mutually independent, the semivariogram and cross-semivariogram are the sum of three components:

4 $${{\rm{\gamma }}}_{uu}({h}_{{\boldsymbol{S}}},{h}_{T})={{\rm{\gamma }}}_{uuS}({h}_{{\boldsymbol{S}}})+{{\rm{\gamma }}}_{uuT}({h}_{T})+{{\rm{\gamma }}}_{uuST}({h}_{ST})$$

5 $${{\rm{\gamma }}}_{vv}({h}_{{\boldsymbol{S}}},{h}_{T})={{\rm{\gamma }}}_{vvS}({h}_{{\boldsymbol{S}}})+{{\rm{\gamma }}}_{vvT}({h}_{T})+{{\rm{\gamma }}}_{vvST}({h}_{ST})$$

6 $${{\rm{\gamma }}}_{uv}({h}_{{\boldsymbol{S}}},{h}_{T})={{\rm{\gamma }}}_{uvS}({h}_{{\boldsymbol{S}}})+{{\rm{\gamma }}}_{uvT}({h}_{T})+{{\rm{\gamma }}}_{uvST}({h}_{ST})$$

The ST lag *h*_*ST*_ is obtained by introducing a geometric anisotropy ratio α: ${h}_{ST}=\sqrt{{h}_{{\boldsymbol{S}}}^{2}+\alpha {h}_{T}^{2}}$. The advantage of the Bilonick model is that it has *S*, *T* and ST components that can be interpreted fairly easily in a physical sense. The disadvantage is that the estimation of the model parameters is challenging. Prior studies estimate the ratio α along with other parameters of the semivariogram^10,11^. In this study, if the ratio αin every semivariogram or cross-semivariogram is estimated, then each αvalue cannot possibly be the same. This outcome may not be in accordance with the physical significance, given that the spatiotemporal ratios for every variable and every pair of co-variables are not the same. In addition, the ratio αis a very important parameter because it determines how to obtain the spatiotemporal distance between two points in space and time; in particular, it determines which observed points in space and time are used as neighboring points when performing ST predictions. If the ratio αdiffers among the semivariograms or cross-semivariograms, different neighboring points will be determined in different variables or pairs of co-variables. Therefore, the ratio α in all of the semivariograms and cross-semivariograms will be considered to be a single parameter.

When models for the semivariogram and cross-semivariogram are obtained, ST cokriging can be performed. The aim is typically to estimate just one variable, which we may regard as the principal or target variable, at a spatiotemporal point *x*_*0*_ (***s***_*0*_, *t*_*0*_) using data that describe that variable and one or more other variables, which we regard as subsidiary variables. The equations used to perform cokriging in the ST domain are exactly the same as those used in standard S cokriging. The equations can be represented in matrix form. For simplicity, we consider only two variables, *u* and *v*. However, the matrices are easily extended to greater numbers of variables. Let **Γ**_*uv*_ denote a matrix of semivariances (including cross-semivariances, in which *u* # *v*) between sampling points in a neighborhood. Let there be *n*_*u*_ places where variable *u* has been measured and *n*_*v*_ places where *v* has been measured. The order of the matrix is *n*_*u *_× *n*_*v*_:

7 $${{\boldsymbol{\Gamma }}}_{uv}=[\begin{array}{cccc}{\gamma }_{uv}({x}_{1},{x}_{1}) & {\gamma }_{uv}({x}_{1},{x}_{2}) & \cdots  & {\gamma }_{uv}({x}_{1},{x}_{{n}_{v}})\\ {\gamma }_{uv}({x}_{2},{x}_{1}) & {\gamma }_{uv}({x}_{2},{x}_{2}) & \cdots  & {\gamma }_{uv}({x}_{2},{x}_{{n}_{v}})\\ \vdots  & \vdots  & \cdots  & \vdots \\ {\gamma }_{uv}({x}_{{n}_{u}},{x}_{1}) & {\gamma }_{uv}({x}_{{n}_{u}},{x}_{2}) & \cdots  & {\gamma }_{uv}({x}_{{n}_{u}},{x}_{{n}_{v}})\end{array}]$$

We denote by **b**_*uu*_ and **b**_*uv*_ the vectors of semivariances for variable *u* and the cross-semivariances:

8 $${{\bf{b}}}_{uu}=[\begin{array}{c}{\bar{\gamma }}_{uu}({x}_{1},{x}_{0})\\ {\bar{\gamma }}_{uu}({x}_{2},{x}_{0})\\ \vdots \\ {\bar{\gamma }}_{uu}({x}_{{n}_{u}},{x}_{0})\end{array}],{{\bf{b}}}_{uv}=[\begin{array}{c}{\bar{\gamma }}_{uv}({x}_{1},{x}_{0})\\ {\bar{\gamma }}_{uv}({x}_{2},{x}_{0})\\ \vdots \\ {\bar{\gamma }}_{uv}({x}_{{n}_{v}},{x}_{0})\end{array}],$$

The matrix equation is then:

9 $$[\begin{array}{cccccccccc} &  &  &  &  &  &  &  & 1 & 0\\  &  &  &  &  &  &  &  & 1 & 0\\  & {{\boldsymbol{\Gamma }}}_{uu} &  &  &  & {{\boldsymbol{\Gamma }}}_{uv} &  &  & \vdots  & \vdots \\  &  &  &  &  &  &  &  & 1 & 0\\  &  &  &  &  &  &  &  & 0 & 1\\  & {{\boldsymbol{\Gamma }}}_{vu} &  &  &  & {{\boldsymbol{\Gamma }}}_{vv} &  &  & 0 & 1\\  &  &  &  &  &  &  &  & \vdots  & \vdots \\  &  &  &  &  &  &  &  & 0 & 1\\ 1 & 1 & \cdots  & 1 & 0 & 0 & \cdots  & 0 & 0 & 0\\ 0 & 0 & \cdots  & 0 & 1 & 1 & \cdots  & 1 & 0 & 0\end{array}]\times [\begin{array}{c}{\lambda }_{1u}\\ {\lambda }_{2u}\\ \vdots \\ {\lambda }_{{n}_{u}u}\\ {\lambda }_{1v}\\ {\lambda }_{2v}\\ \vdots \\ {\lambda }_{{n}_{v}v}\\ {\psi }_{u}\\ {\psi }_{v}\end{array}]=[\begin{array}{c}{{\bf{b}}}_{uu}\\ {{\bf{b}}}_{uv}\\ 1\\ 0\end{array}]$$

The estimated value of variable u at the spatiotemporal point *x*_0_ (***s***_0_, *t*_0_) is then the linear sum:

10 $${\hat{z}}_{u}({x}_{0})=\sum _{i=1}^{{n}_{u}}{\lambda }_{iu}{z}_{u}({x}_{i})+\sum _{j=1}^{{n}_{v}}{\lambda }_{iv}{z}_{v}({x}_{i})$$

The estimation variance is obtained from:

11 $${\sigma }_{u}^{2}({x}_{0})={{\boldsymbol{\lambda }}}^{-1}{\bf{b}}$$

where **λ** is the vector of weights and Lagrange multipliers, and **b** is the right-hand side vector of the matrix equation (9).

### Validation and comparison criteria

The results obtained through the use of STCK with different combinations of co-variables and different numbers of neighborhood points are compared. Soil heavy metals are predicted at each of the sites for which measurements are available using the leave-one-out method, which successively deletes the value of each location where the prediction was utilized. This procedure yields pairs of estimated and observed soil heavy metal concentrations. The root mean squared error (RMSE) is then computed from the pairs of estimated and observed soil heavy metals. The RMSE is defined as:

12 $${\rm{RMSE}}=\sqrt{\frac{{\sum }_{j=1}^{n}\{{[Z({x}_{j})-\hat{Z}({x}_{j})]}^{2}\}}{n}}$$

where *Z*(*x*_*j*_) is the measured value, and $\hat{Z}({x}_{j})$ is the predicted value. Smaller RMSE values indicate greater prediction accuracy.

## Results and Discussion

### Descriptive statistics of soil heavy metals

Descriptive statistics of Cd, Pb, Cu and Zn in the soil samples for each year are presented in Table 1. For Cd, Cu, and Zn, the soil concentrations show steady increases from 2010 to 2014. However, the concentrations of Pb show increases from 2010 to 2013, followed by a small decrease in 2014. Thus, the concentrations of soil heavy metals in the study generally increase over the investigated period.

**Table 1:** Descriptive statistics of the heavy metals in the soils during each year (mg/kg).

Heavy metal	Year	Min	Max	Mean	SD	CV	Skewness	Kurtosis
Cd	2010	1.78	6.22	3.58	0.80	0.22	0.764	0.216
	2011	2.29	6.31	3.68	0.87	0.24	0.822	0.517
	2012	1.98	5.95	3.87	0.85	0.22	0.540	0.555
	2013	2.81	6.91	4.05	0.90	0.22	1.006	0.677
	2014	2.67	6.36	4.02	0.83	0.21	1.119	0.815
Pb	2010	10.91	113.98	27.51	20.24	0.74	2.719	7.362
	2011	11.26	106.63	31.44	24.49	0.78	2.148	3.668
	2012	13.66	125.97	33.01	29.03	0.88	2.328	4.310
	2013	13.11	110.77	35.32	30.89	0.87	1.66	1.028
	2014	12.95	122.63	34.02	31.05	0.91	2.227	3.439
Cu	2010	16.78	98.61	35.46	15.51	0.44	2.111	5.57
	2011	18.87	81.87	38.31	13.35	0.35	1.034	1.428
	2012	22	94.74	39.78	13.42	0.34	1.677	4.806
	2013	18.87	91.77	40.27	15.60	0.39	1.361	1.949
	2014	20.97	103.52	42.91	20.94	0.49	1.754	2.675
Zn	2010	48.05	191.04	84.79	22.54	0.27	0.198	0.203
	2011	50.45	148.61	88.46	21.86	0.25	0.144	0.205
	2012	53.8	200.59	90.50	26.52	0.29	0.120	0.263
	2013	60.39	154.28	93.69	22.24	0.24	0.115	0.199
	2014	57.66	166.75	94.60	23.33	0.25	0.121	0.175

The normality of Cd, Pb, Cu and Zn at all of the sampling points is tested using the Kolmogorov-Smirnov (K-S) method. The K-S test is a nonparametric test of the equality of continuous, one-dimensional probability distributions that can be used to compare a sample with a reference probability distribution (in this case, a normal distribution). This test examines whether two independent distributions are similar or different by generating cumulative probability distributions for the two distributions. The maximum distance or maximum difference is then entered into the K-S probability function to calculate the probability value. Lower probability values (<0.05) means that it is less likely that the two distributions are similar. Conversely, the higher or closer to 1 the value is, the more similar the two distributions are. The results show that the K-S values are 0.001, 0.000, 0.000, and 0.211 for Cd, Pb, Cu and Zn, respectively. Therefore, the Cd, Pb and Cu data were transformed using base 2 logarithms to achieve normal distributions.

### Correlation coefficient analysis

The correlation coefficients were used to determine the relationships among different heavy metals in the soil samples and then to determine the co-variables for each heavy metal while performing the ST interpolation. The Pearson correlation coefficient is a statistical measure of the linear correlation between two variables. This metric takes values between +1 and −1, where 1 indicates total positive linear correlation, 0 indicates no linear correlation, and −1 indicates total negative linear correlation. In this study, Pearson correlation coefficients of the heavy metals in the soil samples are summarized in Table 2. Table 2 shows significantly positive correlations among the four heavy metals. Therefore, the three other types of heavy metals can be treated as co-variables when ST interpolation of one heavy metal is performed using STCK.

**Table 2:** Pearson’s correlation matrix for the heavy metals in the soil samples.

Heavy metal	LogCd	LogPb	LogCu	Zn
LogCd	1			
LogPb	0.358**	1		
LogCu	0.755**	0.244**	1	
Zn	0.593**	0.32**	0.451**	1

^**^Correlation is significant at the 0.01 level (2-tailed).

### The ST semivariogram

**Figure 2:**
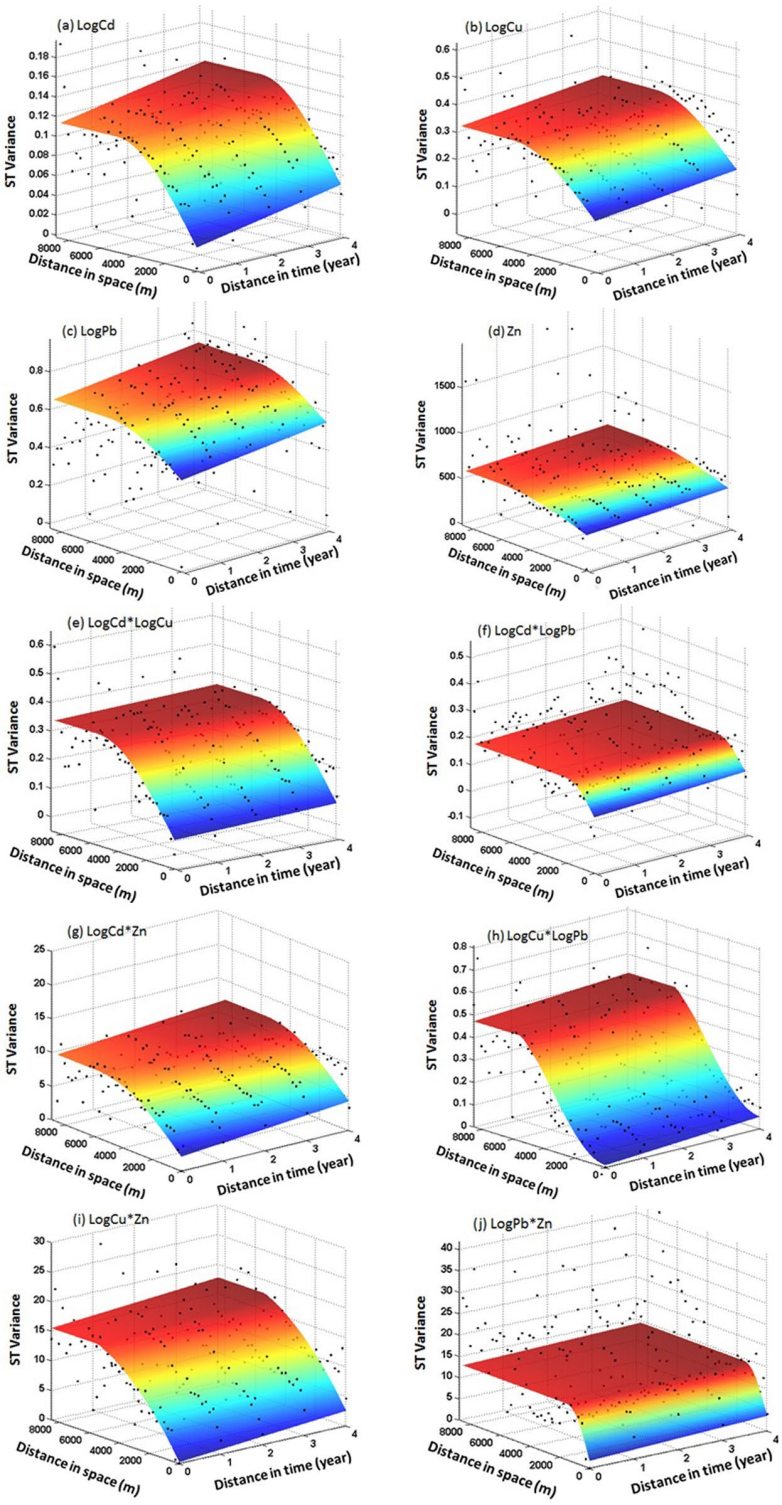
Experimental ST semivariograms and fitting models for LogCd (a), LogCu(b), LogPb(c), and Zn(d) and experimental ST cross-semivariograms and fitting models for LogCd × LogCu (e), LogCd × LogPb (f), LogCd × Zn (g), LogCu × LogPb (h), LogCu × Zn (i), and LogPb × Zn (j).

Figure 2(a–d) shows the experimental semivariograms and fitting models for LogCu, LogCd, LogPb, and Zn. The semivariance displays similar behavior in the space and time directions. In the S direction, the semivariance increases continuously with increasing distance to 5000 to 6000 m and then decreases to approximately 8000 m. All of the semivariograms in the T direction show continuous and slow increases in semivariance for lags of 0 to 4 years. Figure 2(e–j) shows the experimental cross-semivariogram and fitting models for LogCd × LogCu, LogCd × LogPb, LogCd × Zn, LogCu × LogPb, LogCu× Zn, and LogPb × Zn. For LogCd × LogCu (Fig. 2e), LogCd × Zn (Fig. 2g), LogCu × LogPb (Fig. 2h), and LogCu × Zn (Fig. 2i), the cross-semivariance increases continuously with increasing distance to 5000 to 6000 m and is then steady in the S direction. For LogCd × LogPb (Fig. 2f) and LogPb × Zn (Fig. 2j), in the S direction, the cross-semivariance increases with increasing distance to approximately 2000 m and is then steady. In the T direction, all of the cross-semivariograms display continuous and slow increases for lags of 0 to 4 years.

### Model fitting for ST semivariograms and cross-semivariograms

In this case, 4 semivariogram models and 6 cross-semivariogram models are fitted. The types of models are selected based on the figures showing the experimental ST semivariogram and the different logarithmic ST semivariograms. The T parts of all of the semivariograms and cross-semivariograms are modeled using a linear model. The S and ST parts for all of the semivariograms and cross-semivariograms, except LogCu × LogPb and LogCu × Zn, are modeled using a spherical model. The S and ST parts of LogCu × LogPb are modeled with a Gaussian model. The S and ST parts of LogCu × Zn are modeled with an exponential model. A nugget model is used to represent the nugget value of the semivariogram and the cross-semivariograms; hence, the form of the total ST semivariance for LogCu, LogCd, LogPb, and Zn and the form of the ST cross-semivariance for LogCd × LogCu, LogCd × LogPb, LogCd × Zn, and LogPb × Zn are defined as:

13 $${\rm{\gamma }}({h}_{{\boldsymbol{S}}},{h}_{T})={C}_{0}+{C}_{T}{h}_{T}+{C}_{{\boldsymbol{S}}}(\frac{3}{2}\frac{{h}_{{\boldsymbol{S}}}}{{a}_{{\boldsymbol{S}}}}-\frac{1}{2}\frac{{h}_{{\boldsymbol{S}}}^{3}}{{{a}_{{\boldsymbol{S}}}}^{3}})+{C}_{ST}(\frac{3}{2}\frac{{h}_{ST}}{{a}_{ST}}-\frac{1}{2}\frac{{h}_{ST}^{3}}{{{a}_{ST}}^{3}})$$

The form of the ST cross-semivariance for LogCu × LogPb is defined as:

14 $${\rm{\gamma }}({h}_{S},{h}_{T})={C}_{0}+{C}_{T}{h}_{T}+{C}_{S}(1-{e}^{-\frac{{h}_{S}^{2}}{{{a}_{S}}^{2}}})+{C}_{ST}(1-{e}^{-\frac{{h}_{ST}^{2}}{{{a}_{ST}}^{2}}})$$

The form of the ST cross-semivariance for LogCu × Zn is defined as:

15 $${\rm{\gamma }}({h}_{{\boldsymbol{S}}},{h}_{T})={C}_{0}+{C}_{T}{h}_{T}+{C}_{{\boldsymbol{S}}}(1-{e}^{-\frac{{h}_{{\boldsymbol{S}}}}{{a}_{{\boldsymbol{S}}}}})+{C}_{ST}(1-{e}^{-\frac{{h}_{ST}}{{a}_{ST}}})$$

Here, *C*_0_ represents the nugget value of the model; *C*_*T*_ represents the slope of T; *C*_***S***_ and *C*_*ST*_ represents the sill for *S* and ST; *a*_***S***_ and *a*_*ST*_ represent the range parameters of *S* and ST; and

16 $${h}_{ST}=\sqrt{{h}_{{\boldsymbol{S}}}^{2}+\alpha {h}_{T}^{2}}$$

Furthermore, the ratio *α* should be identical in all of the models. The value of *C*_*T*_ is easily calculated using the experimental data. Fitting these 10 models to the experimental data is difficult because 51 parameters must be estimated. We thus use a genetic algorithm to simultaneously estimate these parameters by minimizing a fitness function:

17 $$\sum _{i=1}^{nr\,of\,lags}{w}_{i}{(\hat{\gamma }({h}_{{{\boldsymbol{S}}}_{i}},{h}_{{T}_{i}})-\gamma ({h}_{{{\boldsymbol{S}}}_{i}},{h}_{{T}_{i}}))}^{2}$$

where the weight factor *w*_*i*_ is the quotient of the number of pairs in the lag ${\rm{N}}({h}_{{{\boldsymbol{S}}}_{i}},{h}_{{T}_{i}})$ and the square root of the semivariance $\sqrt{\hat{\gamma }({h}_{{{\boldsymbol{S}}}_{i}},{h}_{{T}_{i}})}$ ^10^. The method of fitting models using a genetic algorithm was introduced and is described in detail in Yang *et al*.^20^. The parameter values resulting from the model fitting procedure are shown in Table 3.

**Table 3:** Parameters of the Bilonick models.

Model	*C*_0_	*C*_*T*_	*C*_***S***_	*a*_***S***_	*C*_*ST*_	*a*_*sT*_	*α*
LogCu	0.17	0.009	0.175	5375	0.22	5734	2085
LogCd	0.033	0.0035	0.103	5843	0.12	4718	
LogPb	0.54	0.032	0.34	5023	0.46	4218	
Zn	503	12.12	296	7445	756	4101	
LogCd × LogCu	0.131	0.006	0.237	4242	0.031	4812	
LogCd × LogPb	0.05	0.0082	0.119	3660	0.187	2159	
LogCd × Zn	0.31	0.12	4.375	4060	12.07	4259	
LogCu × LogPb	0.097	0.011	0.212	2898	0.202	5847	
LogCu × Zn	2.03	0.263	16.48	3203	9.22	6265	
LogPb × Zn	4.53	0.32	5.97	5722	2.75	2511	

### ST interpolation and accuracy evaluation

**Figure 3:**
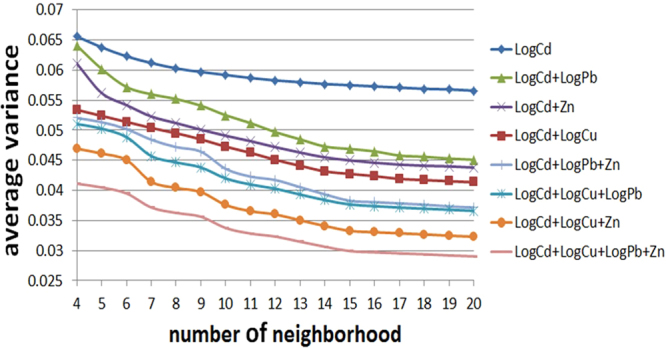
Average variance obtained using kriging with different numbers of neighboring points and different combinations of co-variables.

Based on the methods introduced in section 3.1 and the models of the ST semivariograms and the ST cross-semivariograms (Table 3), STCK is performed for Cd, Cu, Pb and Zn. For example, in the most complex case, Cd is predicted, and all of the other heavy metals, including Cu, Pb, and Zn, are employed as co-variables. The matrix equation (9) is thus extended as follows:

To determine the influence created by the number of neighboring points, we predict the unmeasured ST points using the 4 to 20 nearest ST sampling points around the predicted ST site. The ST distance is determined using formula (16). Because *α* = 2085 and the spatial distances between the sampling points visited in 2014 are between 400 and 1500 m, manyistorical sampling points are incorporated into the group with the nearest ST sampling points. Consequently, the results of STCK are simultaneously influenced by the historical pollution situation and the correlation factors. We also examine the behavior of the STCK prediction using a different combination of co-variables. For example, considering LogCd, the variations in the prediction variance obtained using different numbers of neighboring points and different combinations of co-variables are shown in Fig. 3.

**Figure 4:**
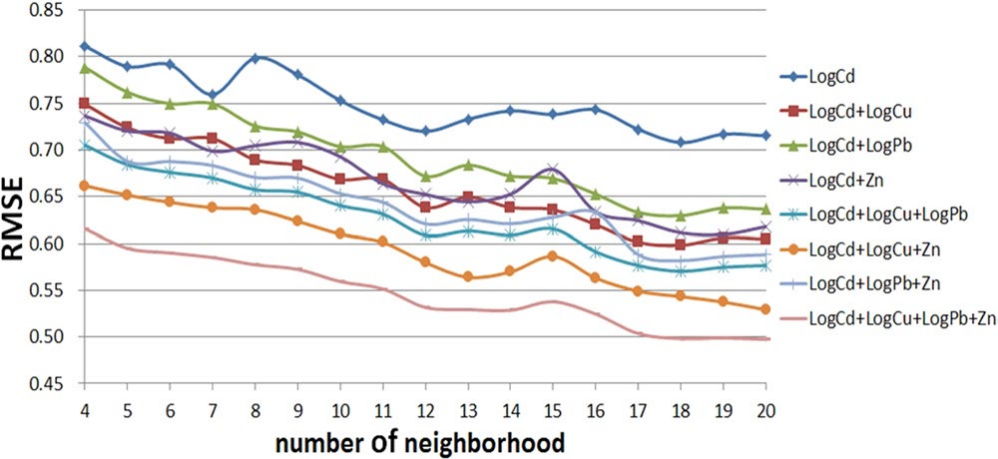
RMSE as a function of different numbers of neighboring points and different combinations of co-variables.

Based on Fig. 3, we conclude that the use of greater numbers of co-variables and neighboring points results in reductions in the variance of predictions. The RMSE cross-validation criterion for Cd in 2014 is provided in Fig. 4. The results of comparing the RMSE are generally consistent with the results obtained for prediction variance. The use of additional co-variables results in reduced RMSE values. However, the use of more neighboring points does not always produce reduced RMSE values. In addition, as shown in Fig. 4, the average RMSE of LogCd + LogCu < the average RMSE of LogCd + Zn < the average RMSE of LogCd + LogPb, indicating that the use of co-variables with relatively high correlation coefficients with the major variable results in greater prediction accuracies than the use of co-variables with relatively low correlation coefficients with the major variable.

**Figure 5:**
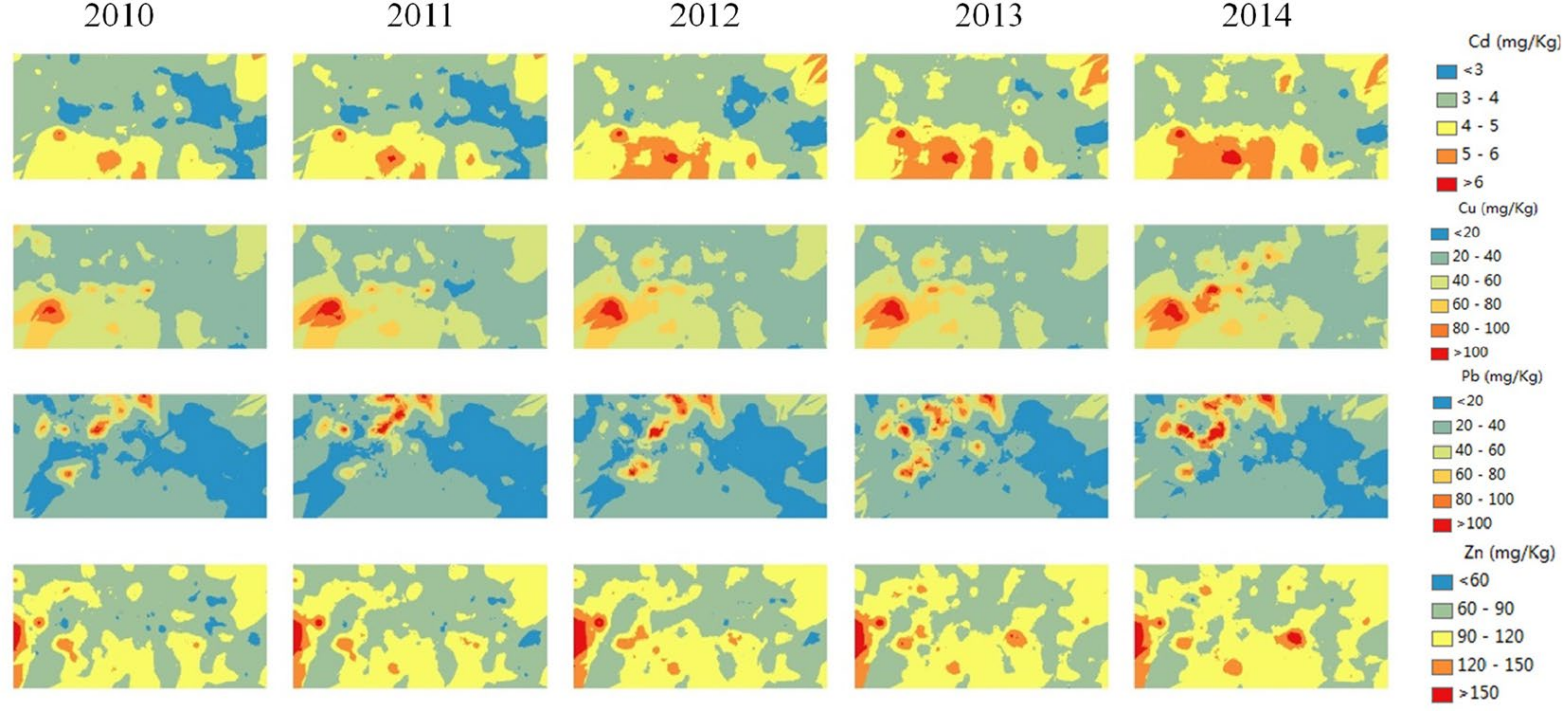
ST distribution of soil heavy metals within the study area (created using ArcMap, version 10.2; http://www.esri.com/).

Figure 5 shows the results of STCK interpolation using three co-variables and 20 neighboring sampling points from 2010 to 2014. The variables, including Cd, Pb, and Cu, are back-transformed to their original scales. Our results reveal a general tendency for elevated concentrations of Cd, Cu and Zn to spread from the southwestern part of the study area to the entire area over time, whereas Pb contamination tends to be concentrated mostly in the northern and western parts. Thus, the ST distributions of heavy metals reveal trends in their ST evolution that can assist in identifying sources of pollution and the directions in which the heavy metals diffuse. For example, based on Fig. 5, we conclude that the sources of the Cd, Cu and Zn pollution are located within the southwestern portion of the study area, i.e., the heavy industrial area of Wuhan City. In addition, the sources of Pb pollution are located within the northern and western parts of the study area.

## Conclusions

In this paper, we present a procedure for carrying out ST predictions of heavy metals based on the STCK method. Soil heavy metals, including Cd, Cu, Pb and Zn, measured in the Qingshan district of Wuhan City in China from 2010 to 2014 are employed as experimental data. The Bilonick model is used to fit ST auto- and cross-variograms, and a genetic algorithm is used to estimate the relevant parameters. The logical ST auto- and cross-variogram models shown in Fig. 2 indicate that the Bilonick model adequately describes the ST variability.

The results of STCK show that the use of additional co-variables improves the ST prediction accuracy; the average RMSE decreases as more co-variables are employed. In addition, the use of co-variables with relatively high correlation coefficients with the major variable results in greater prediction accuracies than the use of co-variables with relatively low correlation coefficients with the major variable. Thus, the use of additional co-variables with relatively high correlation coefficients with the major variable significantly improves the prediction accuracy. In addition, the number of neighboring points affects the prediction accuracy significantly. The use of additional neighboring points results in reduced prediction variance and higher general prediction accuracy.

The results of ST predictions of heavy metals can illustrate trends in ST evolution and can help environmental scientists to infer the locations of pollution sources and the directions in which the heavy metals are diffusing. Suitable environmental governance measures must be proposed.
